# Genome-Wide Association Study Reveals Both Overlapping and Independent Genetic Loci to Control Seed Weight and Silique Length in *Brassica napus*

**DOI:** 10.3389/fpls.2018.00921

**Published:** 2018-07-18

**Authors:** Hongli Dong, Chuandong Tan, Yuzhen Li, Yan He, Shuai Wei, Yixin Cui, Yangui Chen, Dayong Wei, Ying Fu, Yajun He, Huafang Wan, Zhi Liu, Qing Xiong, Kun Lu, Jiana Li, Wei Qian

**Affiliations:** ^1^College of Agronomy and Biotechnology, Southwest University, Chongqing, China; ^2^College of Horticulture and Landscape Architecture, Southwest University, Chongqing, China; ^3^Institute of Crop and Nuclear Technology Utilization, Zhejiang Academy of Agricultural Sciences, Hangzhou, China; ^4^School of Computer and Information Science, Southwest University, Chongqing, China; ^5^Academy of Agricultural Sciences, Southwest University, Chongqing, China

**Keywords:** *Brassica napus*, genome-wide association study, seed weight, silique length, *BnaA.ARF18.a*

## Abstract

Seed weight (SW) is one of three determinants of seed yield, which positively correlates with silique length (SL) in *Brassica napus* (rapeseed). However, the genetic mechanism underlying the relationship between seed weight (SW) and silique length (SL) is largely unknown at present. A natural population comprising 157 inbred lines in rapeseed was genotyped by whole-genome re-sequencing and investigated for SW and SL over four years. The genome-wide association study identified 20 SNPs in significant association with SW on A01, A04, A09, C02, and C06 chromosomes and the phenotypic variation explained by a single locus ranged from 11.85% to 34.58% with an average of 25.43%. Meanwhile, 742 SNPs significantly associated with SL on A02, A03, A04, A07, A08, A09, C01, C03, C04, C06, C07, and C08 chromosomes were also detected and the phenotypic variation explained by a single locus ranged from 4.01 to 48.02% with an average of 33.33%, out of which, more than half of the loci had not been reported in the previous studies. There were 320 overlapping or linked SNPs for both SW and SL on A04, A09, and C06 chromosomes. It indicated that both overlapping and independent genetic loci controlled both SW and SL in *B. napus*. On the haplotype block on A09 chromosome, the allele variants of a known gene *BnaA.ARF18.a* controlling both SW and SL were identified in the natural population by developing derived cleaved amplified polymorphic sequence (dCAPS) markers. These findings are valuable for understanding the genetic mechanism of SW and SL and also for rapeseed molecular breeding programs.

## Introduction

Seed weight (SW) is one of three determinants of seed yield together with seed number per silique and silique number per unit area in rapeseed (*Brassica napus* L.) (Leon, 1993; Ali et al., 1995; Chen et al., 2007). It is a vital breeding objective to increase SW in rapeseed. However, the SW selection efficiency is low in the field, since SW can only be accurately calculated after harvest.

The silique is one of the main photosynthetic organs formed when the leaves fall after flowering in rapeseed (Allen et al., 1971; King et al., 1997). The long silique has a big photosynthetic area and provides more spaces for seed development (Chay and Thurling, 2010). Many studies revealed several overlapped QTLs for SW and SL (Yang et al., 2012; Li et al., 2014; Fu et al., 2015; Liu et al., 2015). A recent study has revealed that *BnaA.ARF18.a* on A09 chromosome controls both SW and SL in rapeseed (Liu et al., 2015). These studies suggest that the length of silique seems to be an ideal indicator of seed weight.

However, the morphological and molecular markers associated with seed weight were seldom applied due to certain problems. For example, the correlation between SW and SL was found to be positive but not very high in several investigations (Knapp et al., 1990; Chay and Thurling, 2010; Yang et al., 2012; Yuan, 2013; Li et al., 2014; Fu et al., 2015; Liu et al., 2015), indicating that the power of predicating SW with SL is not strong. Moreover, in the above-mentioned QTL studies, the genetic variants were only from two parents, and the mapping solution was relatively low with the use of traditional molecular markers, resulting that some linked-markers were not successfully and accurately employed to track SW in the accessions with diverse genetic background. To solve the above problems, it is necessary to understand the genetic control of SW and SL at the whole-genome level.

Association mapping, also called linkage disequilibrium (LD) mapping, studies the statistical association of phenotypes with genetic markers in natural populations (Nordborg and Weigel, 2008; Huang et al., 2010; Xue et al., 2013). In the present study, the significant SNPs in association with SW and SL were investigated in a natural population comprising 157 inbred lines, and the allele variants in *BnaA.ARF18.a* were identified by developing derived cleaved amplified polymorphic sequence (dCAPS) markers. Our results provide valuable information for understanding the genetic control of SW and SL, which may facilitate marker-based breeding in *B. napus*.

## Materials and methods

### Plant materials, field experiments, and trait evaluation

A natural population of rapeseed, comprising 52 spring rapeseeds lines from North America, 54 winter lines from Europe, and 51 semi-winter lines from China (Table S1), was employed to investigate SW and SL. These lines were successively selfed for at least 5 generations prior to further investigation. The lines were grown in the experimental field of Southwest University, Chongqing, (China) during 2013–2016 using a randomized complete block design with two replications. Each plot consisted of 30 plants, with 30 cm between rows and 20 cm within rows spacing. The field management followed the standard agriculture practice.

At maturity, the well-developed siliques in the middle of inflorescence were collected from five individuals in the middle of each plot to investigate the silique length and to calculate the weight using dried seeds.

### Statistical analysis

Analysis of variance (ANOVA) was performed using GLM procedure of SAS^1^. The broad-sense heritability was calculated using the formula, h^2^ = $\sigma_{\text{g}}^{2}$ / ($\sigma_{\text{g}}^{2}$ + $\sigma_{\text{ge}}^{2}$ / n + $\sigma_{\text{e}}^{2}$ / nr), where $\sigma_{\text{g}}^{2}$ is the genetic variance, $\sigma_{\text{ge}}^{2}$ is the interaction variance of the genotype with environment, $\sigma_{\text{e}}^{2}$ is the error variance, and n and r are the number of environments and replications, respectively (Hallauer et al., 1988). Pearson's correlation coefficient between traits of interest was calculated using the SAS CORR procedure.

The best linear unbiased predictor (BLUP) value for each line was calculated across all environments using the mixed linear model by considering both the genotype and the environment as random effects in the R package “LME4” (R Core Team, 2014). The phenotypic evaluation data between 2013–2016 and the BLUP value were subsequently used as a phenotypic data for the association mapping studies.

### Genotyping

Genomic DNA was extracted from the leaf in trefoil stage using CTAB method and sequenced using an Illumina HiseqTM 4000 (Illumina Co, Ltd.) with a 5X sequencing depth and 125-bp paired-end sequencing length. These were mapped to the reference genome *Brassica napus*.annotation_v5 (http://www.genoscope.cns.fr/brassicanapus/data/) with BWA software (http://bio-bwa.sourceforge.net/). SNPs were detected among accessions using GATK software (https://www.broadinstitute.org/gatk/guide/best-practices.php). The SNPs with the missing rate >0.6 were excluded. The remaining SNPs with the missing rate below 0.6 were filled using the software “beagle” v4.1(https://faculty.washington.edu/browning/beagle/beagle.html#download. The SNP loci with heterozygous rate over 25% and minor allele frequency (MAF) less than 0.05 were removed.

### Population structure and genome-wide association analysis

Population structure was analyzed using the software STRUCTURE version 2.3.4 (Pritchard et al., 2000). The program was run with the following parameters: k, the number of groups in the panel varying from 1 to 9; 5 runs for each k value; for each run, both the length of the burning period and number of MCMC (Markov Chain Monte Carlo) replications after burning were set to 10000. The most likely K-value was determined by posterior probability [LnP(D)] and an *ad hoc* statistic Δk (Evanno et al., 2005). Principal component analysis (PCA) based on all 690,953 SNPs was carried out via the TASSEL v5.2 software (Bradbury et al., 2007).

The Q and K matrices were used in the following association analysis using mrMLM (Wang et al., 2016). The *P* (-log_10_*P*) value for each SNP was exported to generate a Manhattan plot using the R package “qqman” (R Core Team, 2014). The input genotype file contained all SNPs that passed through a MAF filter of 0.05. Significant haplotype blocks were identified using the R package “LD heatmap” (Gabriel et al., 2002; Shin et al., 2006).

## Development of dCAPS markers

In order to distinguish the SNPs in association with SW and SL in the natural population with the use of PCR-based molecular marker, the CAPS/ dCAPS primer was designed by dCAPS Finder 2.0 (http://helix.wustl.edu/dCAPS/dCAPS.html), whereas the opposite primer was generated by Primer Primer5 program. The primers and restriction enzymes were listed in Figure 5.

PCR was performed in a total volume of 20 μL, which contained 100 ng of template DNA, 5 μM primer combination, 100 μM of each dNTP, 1 μL 10 × Taq Buffer^+^ (200 mM Tris-HCl pH 8.4, 200 mM KCl, 100 mM (NH4)_2_ SO_4_, 15 mM MgCl_2_), and 1 U Taq DNA Polymerase (Biomed). The reaction system was initially denatured at 94°C for 5 min, followed by 30–35 cycles for PCR amplification, using the following conditions: denaturation at 94°C for 30 s, annealing at Tm-5°C for 1 min, and extension at 72°C for 30 s. The PCR products were digested with the corresponding restriction enzyme at the optimal temperature to activate the enzyme, and the digested products were separated by 3% agarose gel electrophoresis.

## Results

### Phenotypic variations and correlation between seed weight and silique length

**Figure 1:**
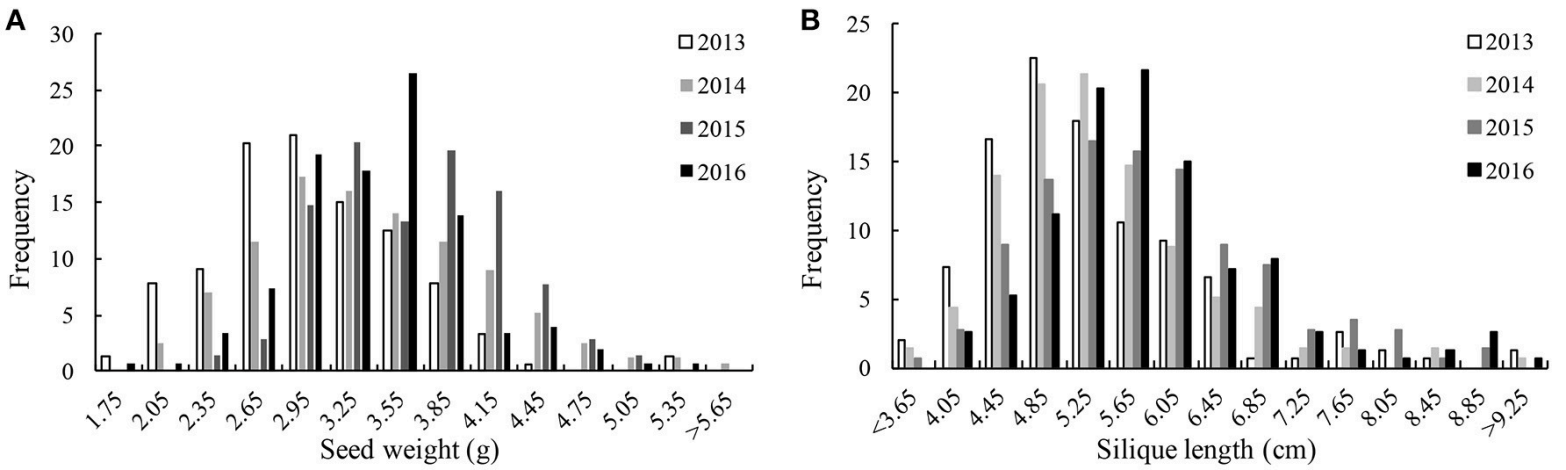
Distribution of seed weight and silique length in 4 years.

We detected significant variations among the 157 inbred lines in 4 consecutive years (2013–2016) for SW ranging from 1.65 to 5.50 g and for SL ranging from 2.97 to 11.44 cm. Both SW and SL exhibited the normal distribution pattern each year (Figures 1A,B), indicating that SW and SL were quantitative traits. Analysis of variance indicated significant differences (*p* = 0.01) among genotypes, environments, and genotype × environment for SW and SL (Table 1). High broad-sense heritability 0.912 and 0.899 for SW and SL, were detected, respectively. It was in accordance with the high correlations between years for SW (above diagonal line) and SL (below diagonal line) (Table 2). However, a weak positive correlation (*r* = 0.332 on average) was also detected between SW and SL in each year (diagonal line) (Table 2).

**Table 1:** Phenotypic variation and analysis of variance (ANOVA) of seed weight and silique length in the association panel.

**Trait**	**Source**	**DF**	**SS**	**MS**	***F*-value**	***P*-value**
Seed weight	Genotypes (G)	153	420.842	2.7506	33.48	< 0.0001
	Environments (E)	3	55.86	18.620	226.66	< 0.0001
	G*E	443	106.817	0.241	2.94	< 0.0001
	Repeatability	2	0.393	0.197	2.39	0.092
Silique length	Genotypes (G)	156	1185.069	7.597	42.09	< 0.0001
	Environments (E)	3	87.989	29.3298	162.5	< 0.0001
	G*E	416	239.951	0.5768	3.2	< 0.0001
	Repeatability	2	0.839	0.420	2.33	0.0984

**Table 2:** Correlation of silique weight (SW) and silique length (SL).

**Environment**	**2013**	**2014**	**2015**	**2016**
2013	0.368^***^	0.81^***^	0.67^***^	0.65^***^
2014	0.69^***^	0.444^***^	0.67^***^	0.58^***^
2015	0.61^***^	0.64^***^	0.212^**^	0.56^***^
2016	0.75^***^	0.76^***^	0.70^***^	0.305^***^

Above diagonal line: correlation for SW between years; Diagonal line: correlation between SW and SL; Below diagonal line: correlation for SL between years

** *Significance level at p = 0.01*,

*** *Significance level at p = 0.001*.

### Genotyping the association population by re-sequencing

The whole-genome sequencing of 157 inbred lines was carried out using an Illumina HiSeqTM 4000 (Illumina Co, Ltd.), producing 45.6 million reads with the length of 125-bp for each line on average (Table S1). A total of 5.29 million SNPs were identified among lines in the natural population (Table S1). After filtering, 690,953 SNPs with high quality were mapped into 19 chromosomes of rapeseed, ranging from 71,147 SNPs in C02 to 16,958 SNPs in A08 (Table S2). The average density of SNP markers across the chromosomes was 10.71 SNP per 10 kb, ranging from 7.56 (C09) to 16.70 (A04) SNP per 10 kb (Table S2).

### Population structure and genetic diversity analyses in natural population

**Figure 2:**
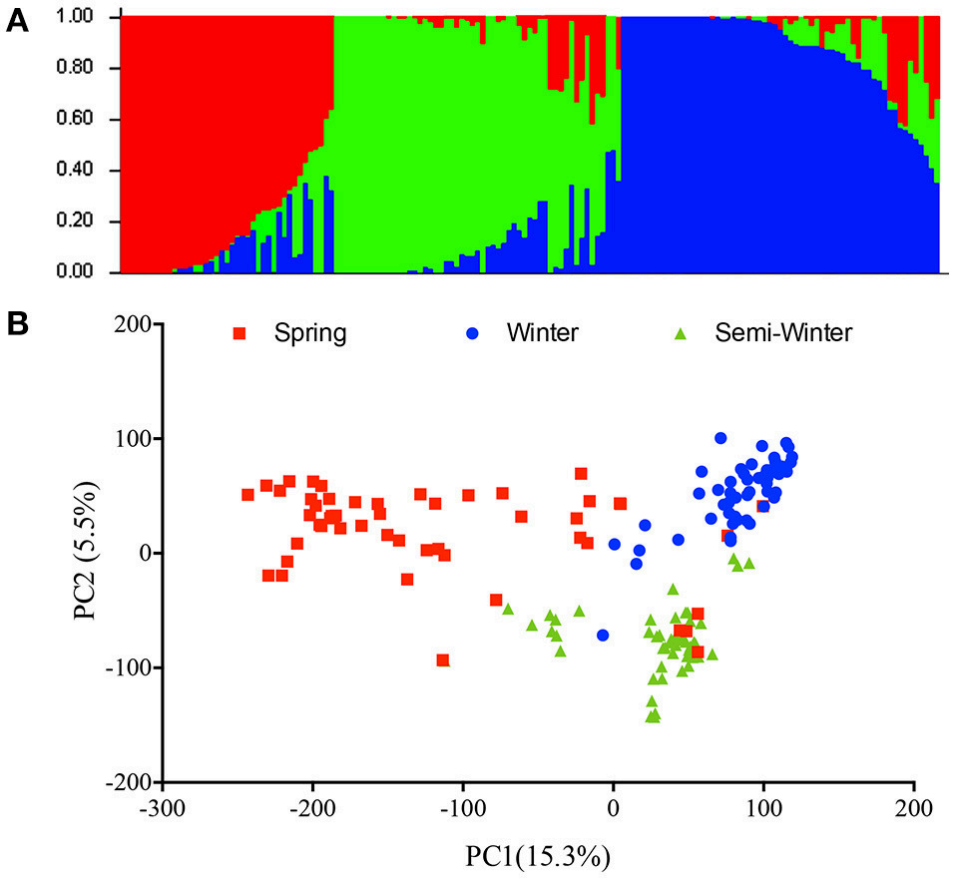
Phylogenetic relationships among 157 inbred accessions. **(A)** Analysis of the population structure composed of 157 rapeseed lines with STRUCTURE. Each individual is represented by a vertical bar, partitioned into colored segments with the length of each segment representing the proportion of the individual's genome, and with the same colored segment representing the same genomic components. **(B)** Principle component analysis. The first two principal components are shown. The individuals with the same label have the same ecotype.

In order to assess the population effect, we analyzed the genetic structure in the natural population using the Evanno method to calculate Δk value and found the highest ΔK at k = 3 (Figure 2A). Therefore, this natural population was divided into three subgroups, which was in accordance with the ecotype habit of accessions, spring, winter, and semi-winter type. This classification was also verified by PCA analysis, where the 157 natural lines were mainly classified into three subgroups (Figure 2B).

### Genome-wide association analysis

**Figure 3:**
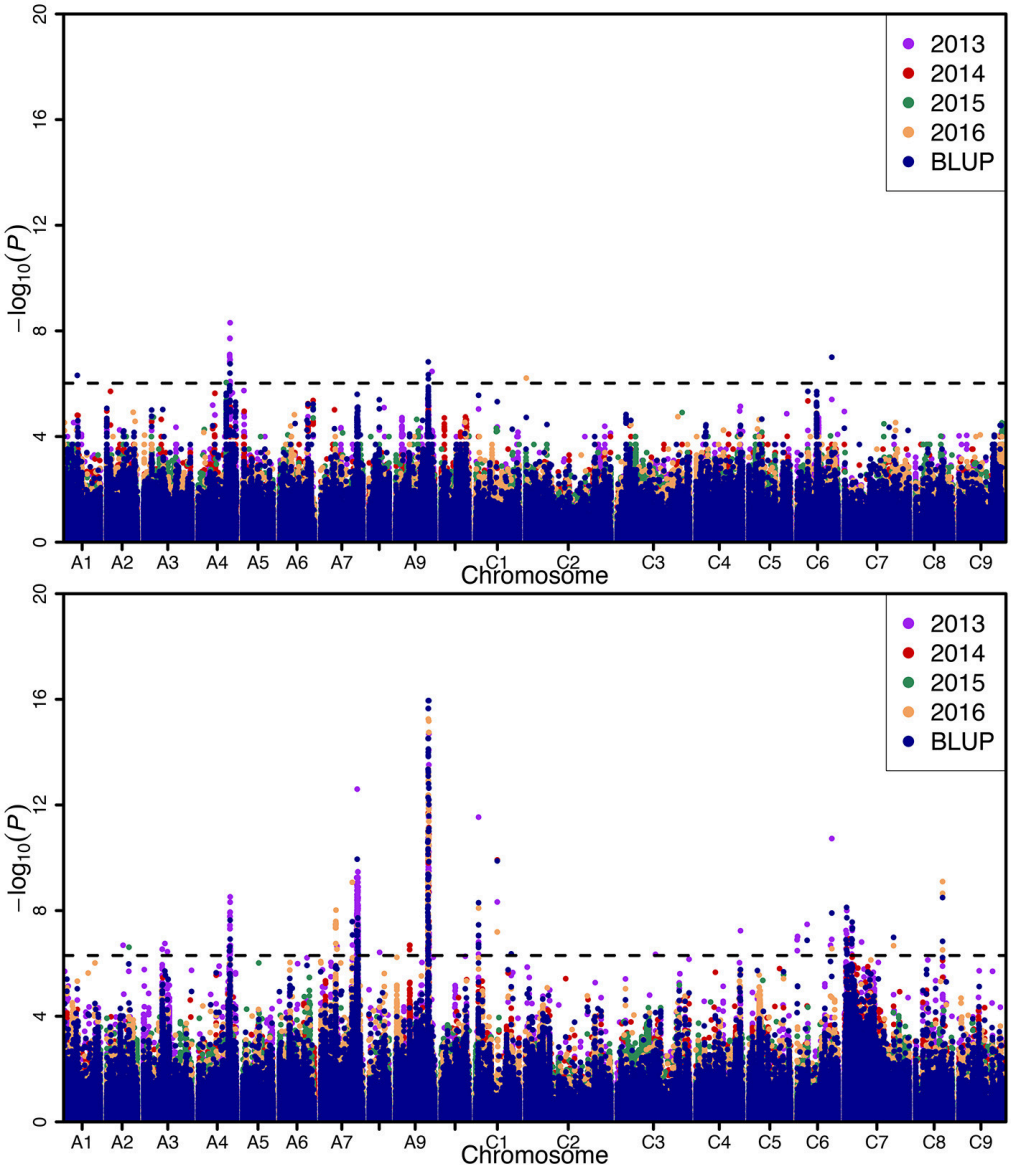
Manhattan plots of the genome-wide association mapping for seed weight (up) and silique length (below). X-axis indicates the physical positions of single nucleotide polymorphism (SNP) along the 19 chromosomes; each environment is displayed by different colors, while y-axis is the value of -log_10_(P). The horizontal dashed line represents the significance threshold with -log_10_(P) = 6.02.

Genome-wide association analysis was carried out using the Q+K model to identify association signals at whole-genome level. In total, we identified 20 SNPs in association with SW on A01 (1), A04 (13), A09 (4), C02 (1), and C06 (1) chromosomes, whereas 742 SNPs in association with SL were identified on A02 (2), A03 (4), A04 (27), A07 (355), A08 (1), A09 (286), C01 (21), C03 (1), C04 (1), C06 (11), C07 (28), and C08 (5) chromosomes using 4 years and BLUP data, with 323 overlapping or linked SNPs for both SW and SL on A04 (39), A09 (282), and C06 (2) chromosomes (Figure 3; Table 3; Tables S3, S4). In comparison with the previous studies (Quijada et al., 2006; Udall et al., 2006; Chen et al., 2007; Samizadeh et al., 2007; Radoev et al., 2008; Shi et al., 2009; Basunanda et al., 2010; Fan et al., 2010; Zhang et al., 2011; Cai et al., 2012; Yang et al., 2012; Li et al., 2014; Fu et al., 2015; Liu et al., 2015; Geng et al., 2016), we found that 13 SNPs on the A04 chromosome were first detected for SW, whereas 447 SNPs were first detected for SL including A02 (2), A04 (27), A07 (298), A09 (7), C01 (1), C04 (1), C06 (9), C07 (28), and C08 (5) (Table 3). The phenotypic variation explained by a single locus ranged from 11.85 to 34.58% for SW and 4.01 to 48.02% for SL, with an average of 25.43% for SW and of 33.33% for SL. Of these, 282 SNPs forming a haplotype block on A09 (27.78–28.61 Mb) were located in the interval of known QTL for SW and SL (Figure 3, Table 3) (Yang et al., 2012; Li et al., 2014; Fu et al., 2015; Liu et al., 2015). Taken together, the overlapping and independent genetic loci control both SW and SL.

**Table 3:** List of single nucleotide polymorphisms with significant association with seed weight (SW) and silique length (SL) using 4 years and BLUP field data among 157 rapeseed lines.

**Code**	**SNP**	***R*^2^%**	**Chr**.	**No**.	**Environment**	**Known loci**
**SW**						
1	8207090	25.23	A01	1	BLUP	Shi et al., 2009; Fan et al., 2010
2	14308857	11.842	A04	1	2015	
3	15814854-15980541	21.13–27.66	A04	12	2013/BLUP	
4	28116249-28180462	31.20–34.58	A09	3	BLUP	Basunanda et al., 2010; Yang et al., 2012; Li et al., 2014; Fu et al., 2015; Liu et al., 2015
5	31325595	23.59	A09	1	2013	Basunanda et al., 2010; Yang et al., 2012; Li et al., 2014; Fu et al., 2015; Liu et al., 2015
6	360417	16.87	C02	1	2016	Udall et al., 2006; Radoev et al., 2008; Basunanda et al., 2010; Fu et al., 2015
7	31182079	28.82	C06	1	BLUP	Shi et al., 2009; Li et al., 2014
**SL**						
1	11025788-17884044	18.26–25.95	A02	2	2013/2015	
2	10984418-14247089	4.01–21.92	A03	4	2013	Zhang et al., 2011
3	15453957-16110806	12.34–34.21	A04	27	2013/BLUP	
4	8153268-8908552	14.85–30.97	A07	53	2013/2016	Zhang et al., 2011;
5	16126618-16380176	23.97–30.17	A07	4	2013/2016/BLUP	Yang et al., 2012
6	19760561-20567053	25.67–39.78	A07	298	2013/2016/BLUP	
7	10476202	32.23	A08	1	2013	Zhang et al., 2011
8	12872271-12949850	30.54–31.26	A09	7	2014	
9	27782829-28612997	15.70–48.02	A09	279	2013/2014/2015/2016/BLUP	Yang et al., 2012; Li et al., 2014; Fu et al., 2015; Liu et al., 2015
10	3750581-3782227	27.73–39.13	C01	16	2013/2016/BLUP	Chen et al., 2007; Cai et al., 2012
11	15053178	27.00–34.42	C01	4	2013/2014/2016/BLUP	Li et al., 2014
12	30151703	24.95	C01	1	BLUP	
13	21910179	14.55	C03	1	2013	Yang et al., 2012
14	46240742	30.88	C04	1	2013	
15	106174-261914	23.42–23.85	C06	2	2013	Zhang et al., 2011
16	1214062-1825439	23.0823.58	C06	2	2013	
17	6231767	22.59–28.12	C06	2	2013/BLUP	
18	30332388-31182079	31.28–43.56	C06	5	2013/2016/BLUP	
19	5380751-5960581	13.96–28.38	C07	25	2013/2014/BLUP	
20	7142765	17.31	C07	1	2013	
21	29121920	6.46–6.76	C07	2	2016/BLUP	
22	31257717-31306490	35.48–38.13	C08	5	2016/BLUP	

### Haplotype analysis of *BnaA.ARF18.a*

**Figure 4:**
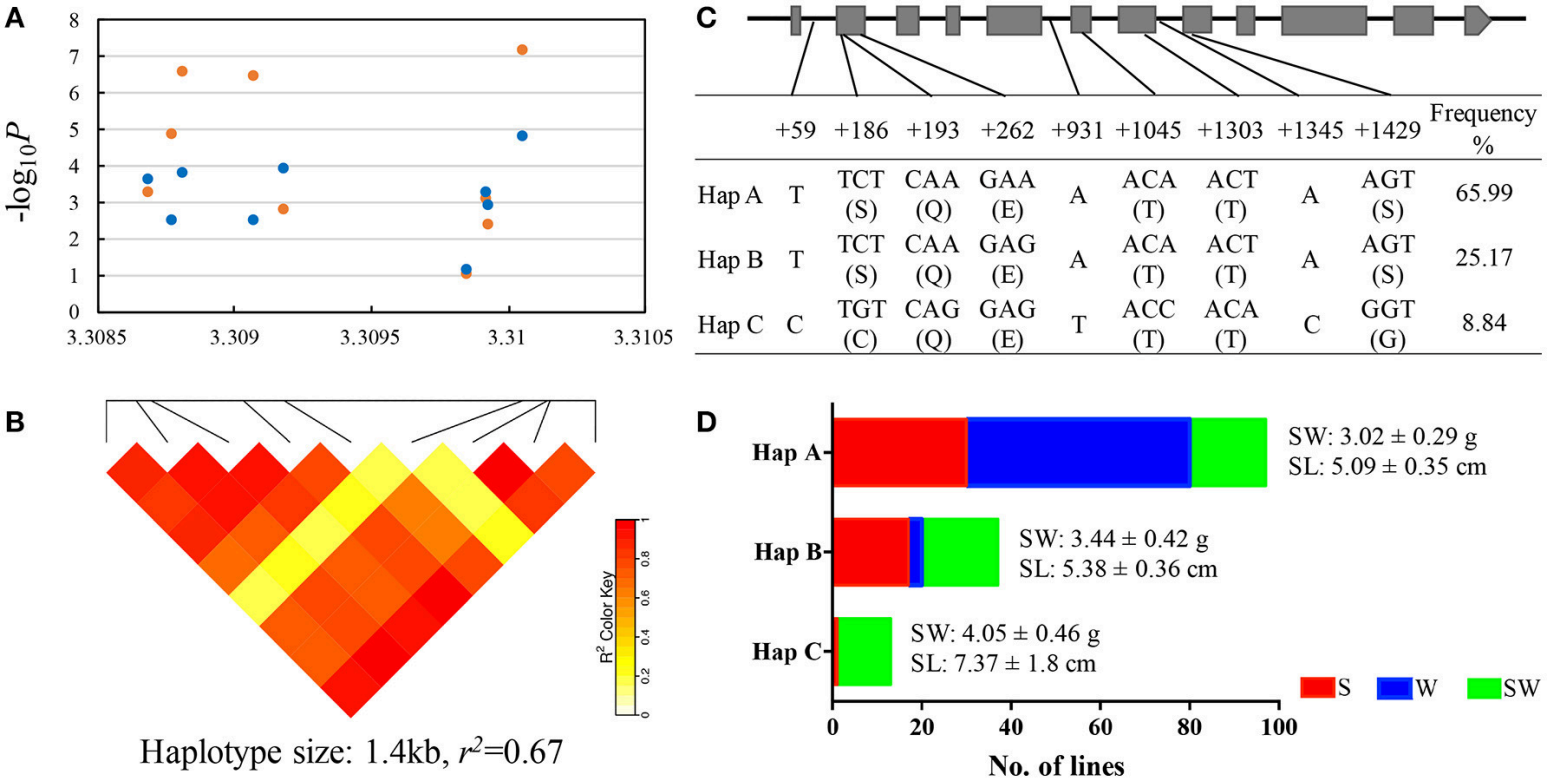
Association mapping and haplotypes of *BnaA.ARF18a* in natural population of rapeseed. **(A)** Plot describing marker-trait association for the trait SW (blue dot) and SL (orange dot) within the gene *BnaA.ARF18.a*. **(B)** Haploblock showing markers in linkage disequilibrium (LD) (>*r*^2^ = 0.6). **(C)** Exon-intron structure of *BnaA.ARF18a* and single nucleotide polymorphisms. Grey boxes and black lines indicated exon and intron, respectively. **(D)** Haplotype analysis for *BnaA.ARF18.a*. in three subgroups. S, W, and SW indicate the spring, winter, and semi-winter rapeseed subgroup, respectively.

We mapped this haplotype block on A09 chromosome to the reference genome of “*ZS11*” (https://www.ncbi.nlm.nih.gov/bioproject/PRJNA394926/) and found a known gene copy *BnaA.ARF18.a* (A09_random: 3307497-3310118 in the “Darmor-*bzh*” reference genome) in this haplotype block controlling both SW and SL in rapeseed (Liu et al., 2015). Within this gene, we detected 6 SNPs (+186, +193, +262, +1045, +1303, +1345) in the exon (we defined the start codon is +1) and 3 SNPs (+59, +931, +1429) in the intron, thereby forming 3 haplotypes (Hap A, B, and C) in the natural population (Figures 4A–C). The 2 SNPs (+186, +1429) in the exon caused the amino acid changes between Hap C and Hap A, B (Figure 4C).

The distribution of haplotypes within the three subgroups is shown in Figure 4D. Significant differences were found among haplotypes for SW and SL. The lines carrying Hap A showed the lowest seed weight (3.02 ± 0.29 g) and shortest silique length (5.09 ± 0.35 cm), followed by Hap B (3.44 ± 0.42 g for seed weight, 5.38 ± 0.36 cm for silique length) and Hap C (4.05 ± 0.46 g for seed weight, 7.37 ± 1.8 cm for silique length) (Figure 4D). In addition, we also found that Haplotype C was not present in winter materials, indicating that introgressing this haplotype into winter material could be very helpful.

### Functional maker development and verification for *BnaA.ARF18.a*

**Figure 5:**
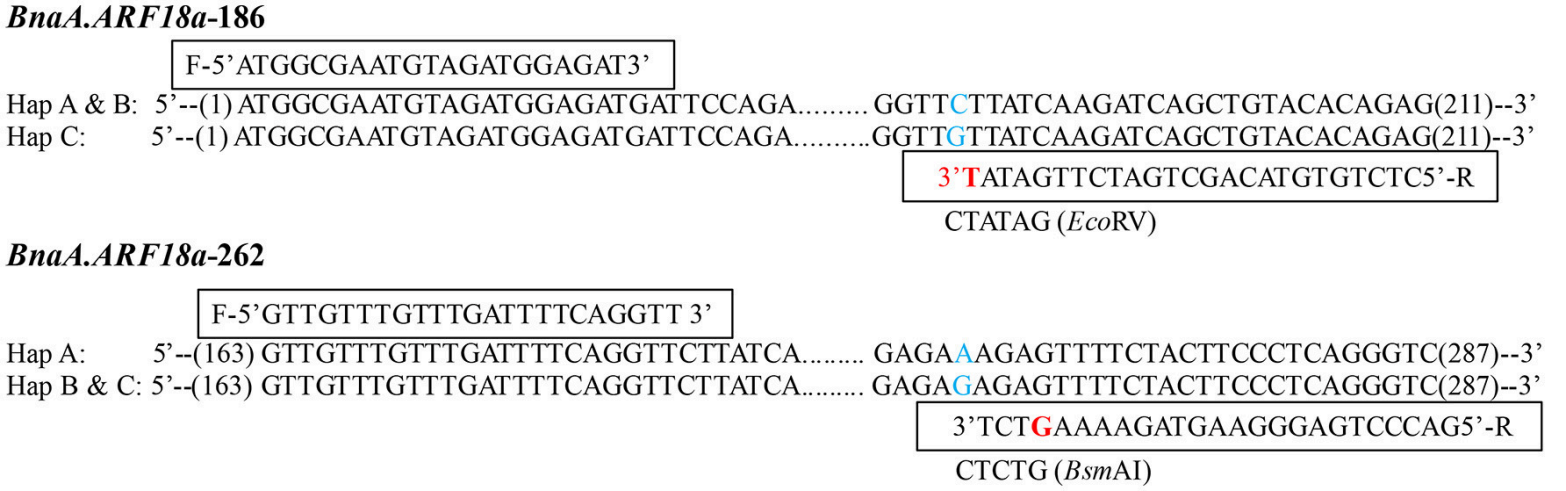
Development of two dCAPS markers in *BnaA.ARF18a, BnaA.ARF18a*-186 (**Top**) and *BnaA.ARF18a*-262 (**Bottom**). The primers and the restriction enzymes were displayed in the box. The blue letter represents the SNP among haplotypes, while the red letter represents the transversion in the restriction site.

**Figure 6:**
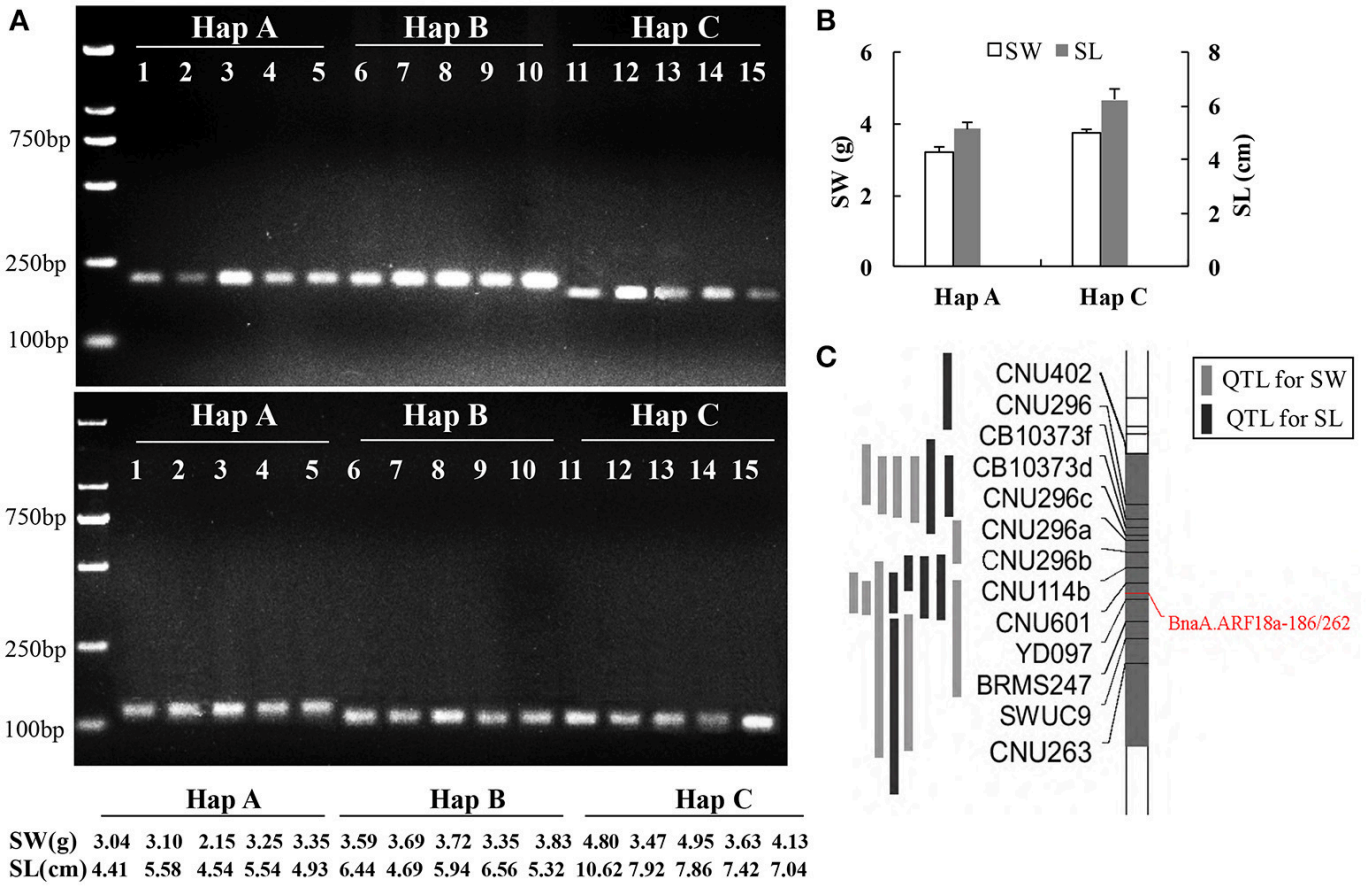
Association of SNPs with seed weight and silique length revealed with dCAPS. Screening accessions in the natural population using *BnaA.ARF18a*-186 (up) and *BnaA.ARF18a*-262 (below) **(A)**; Haplotype analysis **(B)** and location of dCAPS in the interval of QTL for seed weight and silique length **(C)** in a DH population (Fu et al., 2015).

A paralog of *BnaA.ARF18.a* was found on C08 chromosome (30957695-30960363) in the “Darmor-*bzh*” reference genome. In order to distinguish the SNPs among the three haplotypes using a simple and low-cost pattern for crop improvement, we developed two dCAPS functional markers to distinguish the natural variances in the *BnaA.ARF18.a* gene copy specially, and not in its paralog (Figure 5). By transforming A to T at +187 site of *BnaA.ARF18a* gene in the PCR products using “*BnaA.ARF18.a*-186,” the *Eco*RV restriction site (5′…GATATC…3′) was successfully created in Hap C, but not in Hap A and Hap B (5′…GATATG…3′). It means that *Eco*RV can digest the PCR products into two fragments (188 bp and 23 bp) in Hap C, but not in Hap A and Hap B. Similarly, by transiting C to G at the site of +266 of *BnaA.ARF18a* gene, the PCR products using “*BnaA.ARF18a*-262” contained the *Bsm*AI restriction site (5′…GTCTCT…3′) in Hap B and C, but not in the Hap A (5′…GTCTTT…3′). Therefore, the restriction enzyme *Bsm*AI can digest the PCR products into two fragments with the length of 98 and 27 bp in the Hap B and Hap C, whereas the PCR products with the length of 125 bp remain uncleaved in Hap A. The two sets of dCAPS markers were employed to screen the accessions in the natural population. As expected, the PCR products in the accessions with Hap A couldn't be digested by *Eco*RV and *Bsm*AI, whereas those of Hap B could only be digested by *Bsm*AI and those of Hap C could be digested by both the two restriction enzymes (Figure 6A). These results indicated that the three different haplotypes could be distinguished easily with use of the two dCAPS markers.

In the previous study, an overlapping QTL for SW and SL was located in the region from 27.54 to 31.42 Mb on A09 Chromosome in a DH population derived from a cross between “Express” (female) and “SWU07”(male) (Fu et al., 2015), and the reported gene *BnaA.ARF18a* was located in this region. We genotyped two parental lines and DH population using the two dCAPS markers and found that the genotype of “Express” and “SWU07” belonged to Hap A and Hap C, respectively. The two markers were successfully mapped into the interval of known QTLs with co-segregation pattern (Figure 6C). The seed weight and silique length in the DH lines with the allele from “Express” were significantly lower than that of “SWU07” (*P* < 0.001) (Figure 6B). These findings reveal that the set of dCAPS can be used to identify the optimal allele controlling both the SW and SL in A09.

## Discussion

Seed weight is one of the most important yield traits and is determined by at least five signaling pathways in *Arabidopsis* and rice (Li and Li, 2016). In the previous studies, approximately 120 QTLs have been identified for SW in rapeseed (Quijada et al., 2006; Udall et al., 2006; Radoev et al., 2008; Shi et al., 2009; Basunanda et al., 2010; Zhang et al., 2011; Yang et al., 2012; Li et al., 2014; Fu et al., 2015; Liu et al., 2015). In the present study, a natural population of rapeseed was genotyped by whole-genome re-sequencing, producing 690,953 informative SNPs. Of which, 20 and 742 SNPs were detected to associate with SW and SL, respectively, including more than half of loci, which were previously reported. To our knowledge, this is the first time that genome re-sequencing has been used to dissect genetic loci controlling SW and SL in rapeseed. Our data demonstrate the power of this approach in dissecting agronomic traits.

The *BnaA.ARF18.a* gene (auxin-response factor 18) was reported to control SW and SL in a segregation population that derived from a cross between big seed parental line “zy72360” and small seed parent line “R1” (Liu et al., 2015). This gene was located in the known QTL interval on A09 chromosome (Yang et al., 2012; Li et al., 2014; Fu et al., 2015; Liu et al., 2015). We analyzed the haplotype of “zy72360” and “R1” using five SNPs (+262, +1045, +1303, +1345, +1429) within the *BnaA.ARF18.a* reported by Liu et al. (2015) and classified “zy72360” and “R1” into the Hap A (big seed group) and Hap C (small seed group), respectively. It showed the validity of the haplotype analysis in this study. Moreover, the successful development of the two functional markers to distinguish the natural variances of the *BnaA.ARF18.a* gene will be helpful for rapeseed molecular breeding.

The silique is one of the main photosynthetic organs in the absence of leaves, contributing 80–95% of photosynthetic products in rapeseed during seed filling (King et al., 1997; Hu et al., 1998; Bennett et al., 2011), whereas it is one of the storage organs by transferring energy into the developing seed. Meanwhile, the silique is one of energy-consuming organs owing to its own development. Although the long silique of rapeseed has wide photosynthetic area to potentially produce more energy, it consumes a lot of energy for its own development. In this research, we detected a few overlapping loci that controlled both SW and SL, explaining no more than 34 and 48% of genetic variance of SW and SL, respectively. It was in accordance with a weak positive correlation between the length of silique and seed weight (Basunanda et al., 2010; Yang et al., 2012; Li et al., 2014; Fu et al., 2015; Liu et al., 2015). This suggests that the optimal length of silique is needed to balance the processes of producing, transferring, and consuming energy in silique in order to produce big seed size. Our data suggested that the overlapping and independent genetic loci controlled SW and SL, respectively. It is possible to improve seed weight without overdepending on long silique.

## Data availability

The data set generated and analyzed in the current study is available from NCBI under BioProject accession PRJNA358784.

## Author contributions

WQ conceived the study. WQ and HD designed the experiments. HD, YiC, KL, JL and DW performed the genotyping of the association panel. HD, CT, YL, SW, YaC, DW and YanH participated in the phenotyping of seed weight and silique length. HD, CT, YF, YajH and YaC participated in the linkage mapping experiment. HD, QX, CT, HW, ZL and WQ analyzed the data. HD and WQ wrote the paper. All the authors have read and participated in the editing of the manuscript.

### Conflict of interest statement

The authors declare that the research was conducted in the absence of any commercial or financial relationships that could be construed as a potential conflict of interest.

## Footnotes

^1^http://support.sas.com/software/93/
